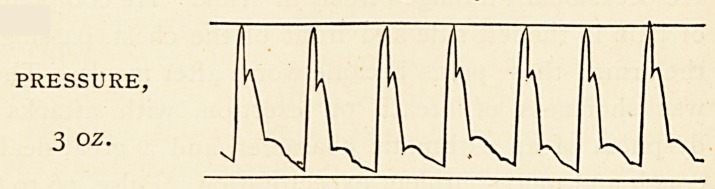# Notes of Out-Patient Cases

**Published:** 1888-06

**Authors:** J. Michell Clarke

**Affiliations:** Assistant-Physician to the Bristol General Hospital; Assistant-Lecturer on Physiology at the Bristol Medical School


### NOTES OF OUT-PATIENT CASES. By J. Michell Clarke, M.A., M.B. Camb., M.R.C.P. Lond.; Assistant-Physician to the Bristol General Hospital; Assistant-Lecturer on Physiology at the Bristol Medical School.

### SOME SPINAL CASES.

J. B., an exceptionally powerful and well-built man, 32 years of age, of healthy family, complained of loss of power in both legs. He had lately been engaged in deep-sea diving at a depth of 125 feet below the surface. He had always been healthy, had never had syphilis, but occasionally when off duty indulged to excess in alcoholic liquors. He had several times lost the use of his hands for about an hour after coming out of the water. The present illness began, about three months ago, with numbness in both feet, feeling as if he had several pairs of stockings on, or was walking on a thick carpet. Since then weakness in both legs came on and gradually increased, so that he could no longer walk any distance, and was obliged to give up his work from inability to mount a ladder. He is very giddy in the dark, and cannot walk without watching his feet, as, if he stumbles against anything, he falls down and is unable to recover himself. He has never had the least pain, nor any attacks of sickness. On walking he dragged his feet along the floor with a scraping noise, and on attempting to walk fast there was marked ataxy; he had great difficulty in turning round; and on placing his feet together and closing his eyes, fell backwards if not supported.

On examination there was no wasting or rigidity of the leg muscles; the superficial reflexes were everywhere normal; the knee-jerk was distinctly present, but not active; the bicipital and tricipital tendon-reflexes were exaggerated. No loss of sensation to touch or pain could be detected, but there was some loss of muscular sense in the legs, as he did not appreciate well the change of position when his legs were passively moved. There were no paralyses. The pupils were equal, and acted both to light and accommodation. He was taken into the Hospital, and after lying in bed for some weeks he gained strength in the limbs, but the symptoms were otherwise materially the same. The functions of micturition and defecation were not affected.

Divers, as in the above case, sometimes suffer from paraplegic symptoms, which come on generally from half an hour to an hour after they emerge from the water. As a rule only those who work at considerable depths below the surface suffer. Less severe symptoms are common among them, especially a sensation of numbness and pricking in the feet and lower part of the legs, as if they had "gone to sleep:" this affection is known amongst them as "the bends." The interesting feature of the above case was the preponderance of symptoms referable to injury of the posterior columns of the cord over the paraplegia, which gave the case considerable resemblance to tabes dorsalis, differing from the latter affection, however, in the presence of the knee-jerks, absence of lightning pains and of "crises," and of implication of the pupils. The affection of the spinal cord is doubtless produced by the high atmospheric pressure at which divers work, but in what particular way is not known. In two cases (Leyden, Schultz) quoted by Gowers, no hæmorrhages into the cord were found, but in each there was slight disseminated myelitis in the dorsal region. Moxon attributed the effect to the imperfect blood-supply to the lumbar portion of the cord; but Gowers points out that the dorsal, and not the lumbar, portion of the cord seems to be chiefly affected.

The following is one of the rare cases in which damage to the cord, not the result of injury, is entirely confined to one side. The lesion, from the suddenness of the onset and exceedingly limited distribution of the mischief, was probably a hæmorrhage. The patient, a labourer of 44 years of age, had a sudden attack of pain in the back after doing some heavy work, with loss of power in the right leg: this weakness in the right leg has continued ever since. He was a tall, thin, healthy-looking man; had had syphilis and an attack of ague when a young man, but no other illness. He used to drink too freely, and to be careless about exposure to cold. The thoracic and abdominal organs were healthy, but the arteries showed marked thickening of their coats. He suffered from constipation and slowness of micturition. The muscles of the right leg and calf were somewhat rigid and showed fibrillar tremblings; there was no obvious wasting of individual muscles, but the right leg measured one inch less than the left in circumference. The muscles of the right leg reacted to weaker constant and interrupted currents than the left. There was no loss of sensation. Ankle- and knee-clonus was readily obtained on the right side, but on the left there was no clonus; the knee-jerk was much exaggerated on the right, and brisk on the left, side. The plantar and gluteal reflexes were absent on the right side, but present on the left, though the gluteal was only obtained with difficulty there. The epigastric, lower abdominal, and cremasteric reflexes were normal on both sides—no tenderness or other abnormality was observed over the spinal column. I saw him about eighteen months after the first visit, and the symptoms remained unchanged. The above signs indicated that the lesion was situated on the right side of the cord in the upper lumbar region.

The following are two well-marked cases of pseudo-hypertrophic paralysis in two brothers in the early stage of enlargement of muscles. There was no instance of nervous disease in the family on either the father's or the mother's side, and no other case of the kind in her own children according to the mother's statements. At my request, however, she brought the youngest boy to me, and he was found to be also affected. The first patient is a boy 8 years old; his parents are healthy, except that his mother had acute rheumatism four years ago. His grandfather and an aunt on the father's side died of phthisis. He has two brothers and three sisters: one brother æt. 22, three sisters æt. 19, 11, 7 are all quite strong and healthy; the youngest brother, æt. 5, is also affected. No history of syphilis in the parents could be obtained. He was born two weeks before the proper time, delivery was easy; he cut his teeth without any disturbance of the general health, and has never had any illness. He was unable to walk until three years old, and inclined to fall down when placed on his legs; he has always lived in a healthy rural district of Somerset. He now complains of weakness in walking, of inability to walk any distance, and of liability to stumble and fall down, with difficulty in getting up again. He is intelligent, does well at school, and looks healthy.

He stands with the shoulders well thrown back, the head forwards, the buttocks prominent, an exaggerated concavity over the lower dorsal and lumbar spine, with the stomach thrown forward and the feet widely apart. The gait shows the characteristic oscillation from side to side at each step: and in rising from the sitting posture he first gets upon his hands and knees, then places his hands upon the thighs just above the knees, and raises them higher and higher upon the thighs, and by this means gets the trunk into the erect posture. All his movements are weak, and he finds considerable difficulty in going upstairs.

The muscles of the calves are greatly increased in size, and the flexors of the knee show next to them the greatest increase; the glutei are also enlarged, but very much less so in comparison with the above. The vastus externus shows enlargement, but the recti are smaller than normal. To the feel the hypertrophied muscles are firm, hard, and resistant. The lumbar muscles seem weak and not well developed. The infra-spinati show great enlargement: the latissimus dorsi on each side is wasted, and there appears to be also wasting of the serratus magnus. Only the upper part of the pectoralis major is present, the lower being atrophied. The normal folds of the axillæ are therefore almost entirely absent. The atrophied muscles hardly respond at all to electrical stimulation; the enlarged muscles give diminished reaction to both the constant and interrupted currents. Both the superficial and the tendon-reflexes are sluggish and diminished in amount. The curving forwards of the lumbar spine disappears in the sitting posture, the whole spinal column then presenting a curve with the concavity forwards. When he stands, a vertical line from the scapula falls about an inch behind the buttocks. There is no loss of sensation; no giddiness; no affection of the special senses, and he walks well with his eyes shut. The muscles of the arm and forearm are not affected. The case well shows the ordinary features of the disease, especially in the marked affection of the infra-spinatus, latissimus dorsi, and pectoralis major; and I have recorded it as being a good example of a somewhat rare, though now well-known, affection.

In the second case, the younger brother of the above, æt. 5, the mother had noticed nothing wrong, and stated that he was always healthy but was very late in learning to walk, not walking till three years of age. On examination he is a healthy-looking boy, well nourished, runs and walks about well, is restless and active, readily jumping up when laid upon his back. He stands and walks, however, with his legs rather wide apart, and on examination it is seen that the calf-muscles are enlarged, hard, and firm; there is also considerable enlargement of the flexors of the knee, and of the recti femoris and vasti externi. The glutei show less increase in size and are rather flabby. The pectoralis major is distinctly atrophied in its lower part, but not nearly to such a marked extent as in the elder brother: the latissimus dorsi is small, but shows no definite atrophy. The infra-spinatus is somewhat enlarged, more especially on the right side, but the increase in size is not great. The other muscles are normal. The arms are not affected. The knee-jerk is brisk on both sides, the superficial reflexes normal: the muscles react fairly to electrical stimulation of both kinds, but the enlarged muscles somewhat slowly.

The flexors of the knee do not very commonly show any marked increase in size in this disease—in both these cases it was very well marked, and with the picking out of the vastus externus formed a type common to the two which may indicate a family vulnerability of these muscles. In a large family one nearly always finds more than one child is affected, and the second case shows the importance of not forming a negative conclusion from hearsay evidence even after the most careful anamnesis, since, until movement becomes markedly interfered with, the changes produced by the disease are not sufficiently striking to be noticed by the lay eye. The mother was most positive in this instance that the other children were unaffected.

### DISEASE OF PULMONARY AND AORTIC VALVES, THE LESION OF THE PULMONARY BEING MOST SEVERE.

The patient was a healthy-looking man, of 40 years of age, with a somewhat anxious expression: he was a sawyer by trade. He was first seen on January 23rd, and complained of shortness of breath and swelling of the legs, with pain and tightness of the chest. The illness began, four months previously, with a severe cold, before which time he had always been strong and healthy. On examination, the lungs were healthy, and the liver slightly enlarged. The cardiac impulse was heaving in the sixth space, two inches outside the nipple line, and there was pulsation over the fifth, sixth, and seventh spaces. A double thrill was felt at the base. The cardiac dulness began above at the third rib, and extended laterally from the right border of the sternum to about 1½ inches outside the nipple-line. A blowing systolic murmur was heard over the aortic cartilage, conducted upwards, and also a soft, blowing, diastolic murmur, conducted down the left side of the sternum, possessing the usual characters of an aortic regurgitant murmur. The second sound could also be distinguished, occurring with the diastolic murmur at the base. At the junction of the third cartilage with the sternum was heard a very musical, squeaking sound, occurring towards the end of the systole, and conducted towards the left for a short distance both upwards and downwards. At the apex was a rough, blowing, systolic murmur, audible in the axilla and at the angle of the left scapula. The diagnosis made was aortic stenosis and regurgitation and mitral regurgitation. A month later the same murmur was heard at the apex, and diastolic murmur at the base : the systolic murmur at the base was very much rougher, and a note was made that it had the characters of a friction-sound ; the second sound was reduplicated, and occupying the latter third of the first sound was the same musical squeak, unaltered in character, but louder, and now heard over the greater part of the cardiac area of dulness, but not to the right of mid-sternum.

As the man grew worse, he was admitted, under the care of Dr. Markham Skerritt, who has kindly permitted me to use the results of the *post mortem* examination. He died on May 24th, the sounds remaining as above described.

At the autopsy the heart was much enlarged, weighing thirty ounces; both the right auricle and ventricle were greatly dilated, and also hypertrophied, and the pulmonary and tricuspid valves incompetent. On each segment of the pulmonary valve was an exuberant mass of granulation tissue, but no ulcerative changes; the granulation masses were about the size of one to two peas, occupying the free borders and contiguous part of the ventricular aspect, and filling up the orifice when the valve was closed. The granulations were very irregular in form, and on one segment a portion, about $\frac{1}{8}$ inch in length, attached only at one end, must have floated freely in the blood-current; they were for the most part elastic and soft, firmly attached and not friable, but in the deeper parts were hard and partly calcified. There was a small atheromatous patch on the inner lining of the pulmonary artery at its commencement. The cavities of the left side of the heart had also undergone great dilatation, and there was much hypertrophy of the left ventricle, the muscle-tissue being healthy. The mitral orifice was enlarged and incompetent, admitting easily five fingers; the valve-segments were healthy. The aortic valve was also incompetent; and on each segment was a small collection of granulation tissue, about the size of a split-pea or smaller, and occupying the centres of the free borders of the valves, harder than that on the pulmonary valve-segments, and appearing to be of older date.

On the upper part and anterior surface of the right

VEGETATIONS ON PULMONARY VALVES.
(A little larger than natural size.)

ventricle was an elongated patch of firm, but not hard, white fibrinous material from old pericarditis, about one inch in length and $\frac{1}{3}$ inch in breadth. The pericardium contained a few ounces of fluid, and there were no adhesions. There was fluid in the pleural and peritoneal cavities, and the organs showed the usual lesions of chronic congestion.

The pulse, of which a tracing is appended, was of the water-hammer character, the dicrotic wave being feebly marked, but the arteries remaining fairly full between the beats.

PRESSURE,

3 OZ.

In the presence of aortic valve-disease, and of the patch of pericarditis above mentioned, it is difficult to know which of the above murmurs could be attributed to the lesion of the pulmonary valve. I am inclined, however, to attribute the musical, squeaking sound heard at the left third cartilage to the obstruction offered to the passage of the blood by the pulmonary valvulitis, from the fact that it was heard at the first examination; while, at the second examination, the presence of a very rough systolic murmur, like a friction-sound, was noted at the base, which had not previously been heard, while the musical murmur had increased in intensity and was heard over a wider area. We may suppose that in the interval between the two examinations the pericarditis had occurred, while the pulmonary granulations had increased, and so gave rise to a louder murmur. The very musical character of the murmur, the way in which it was conducted, and its position of the greatest intensity at the third left cartilage, are also in favour of its origin at the valve, whilst this situation is higher than that of the roughened patch on the heart's surface.

### ANEURISM OF FIRST PART OF AORTA. SUDDEN DEATH FROM RUPTURE INTO THE PERICARDIUM.

The patient was a labourer, 58 years of age; a tall, strong man, rather anæmic and pasty-faced. Except for an attack of pleurisy in the left side six years ago, he had had no illness; since then, he suffered from winter-cough. He occasionally indulged freely in drink. He complained of pain in the left side and front of the chest, passing to the arms: these pains became worse after meals. There was shortness of breath on exertion, with attacks of dyspnœa of an asthmatic character, and a great deal of cough and muco-purulent expectoration. Pulse, 96 to the minute, weak, equal in both wrists.

On examination, the chest presented the usual signs of well-marked emphysema, and there was much tenderness over the sternum and front of the chest. The heart-sounds were normal; the pulmonary second sound accentuated, and its impulse in the epigastrium.

The liver was not enlarged. The arteries were everywhere markedly atheromatous, and the urine contained no albumen. He was ordered first a bismuth mixture, and then one containing potass. iodid. and extract. stramonii. He very much improved, and returned to work, apparently relieved of his symptoms, six weeks after he first came under my care. Three days later he came home drunk, sat up in bed in the night with a gurgling noise in the throat, and fell back dead. At the autopsy, the pericardium was found full of blood and recent clot; and a globular aneurism, about the size of an orange, was found arising from the posterior aspect of the first part of the aorta, just above the sinuses of Valsalva. It projected directly backwards, immediately beneath the bronchi and main branches of the pulmonary artery, so that it had not exercised pressure on any of the surrounding structure. It was entirely limited to the first part of the aorta. The inner surface of the aneurism was exceedingly atheromatous, and on the right side perforation had occurred in the base of an atheromatous ulcer into the pericardial sac, immediately under its upper fold. The aortic valves were a little thickened and stiff, but competent, and the other valves were healthy, the heart being of the normal size.

There was emphysema of both lungs, especially marked along the anterior borders; the other organs were normal.

The case shows, like many others, that an aneurism in this situation may attain a fair size with a complete absence of physical signs, especially when there is concomitant pulmonary emphysema; and, since the most common termination is rupture into the pericardial sac, that this rupture, and consequent sudden death of the patient, may be the first sure indication of the aneurism. Attacks of dyspnœa of asthmatic character are always suspicious of aneurism, when they come on in elderly persons for the first time, especially when attended by pain in the region of the heart.

### CASE OF "ADULT CHOREA," WITH SOME SYMPTOMS OF PARALYSIS AGITANS.

The patient is a man, 64 years of age, who has been under observation now for nearly two years, without any change in the symptoms. He comes of a healthy stock. and, except for winter-cough during the last two years, has always enjoyed good health.

He was a nailmaker by trade, but was obliged to give it up thirty-three years ago. Five or six years before that time he began to suffer from choreiform movements, which began in the left wrist, spread to both shoulders, and then to the right arm, neck, and head; at the same time the arms became weak. The movements interfered very much with his work, and thirty-three years ago the weakness increased suddenly in the right arm, so that he lost the use of it, and was never able again to make nails. After six weeks, however, he found he could do ordinary labour, and continued at work until fourteen years ago, when the weakness in the arms, shoulders, neck, and hands had increased so much as to entirely prevent it, Since that time the movements and loss of strength have steadily increased, though with extreme slowness. The patient now stands in the attitude of paralysis agitans, with an anxious, fixed, mask-like expression, the head carried forwards, the shoulders and back rounded and bent, the arms carried in front of him, flexed at the elbows; the fingers flexed at the metacarpal joints, and somewhat in position for holding a pen, but with no rolling movement of thumb on fingers.

All muscular movements are slowly performed, and in a weak and jerky manner ; and the speech is monotonous.

There are constant, rather slow, choreiform movements of the head ; the face is turned first to one side and then to the other, and at the same time directed upwards or downwards ; or the face is raised to look directly upwards, the chin being pushed out forwards. Or, again, the head is pulled downwards directly towards the shoulder, either to the right or left, or the chin is depressed upon the sternum. At the same time there are irregular movements of the shoulders and arms, consisting of both shoulders being shrugged upwards, or the point of either the right or left shoulder raised alone ; in the arms, flexion and extension at the elbow-joint occur, the hands being now raised, now depressed. These movements are continually going on, even during sleep: when he uses his arms or hands, the movements of the head are increased. When first seen, some two years ago, fine tremor of the left forearm, with fibrillar twitching of muscles, was noted, but this has not been observed since. There is slight tremor of the head. The compensatory movements of the eyeballs during the motions of the head give almost the appearance of a slow nystagmus. On extending the arms, some tremor of a jerky character supervenes, the grasp of both hands is very weak, the left wrist is slightly dropped, and he is unable to carry anything in his left hand. The muscles of the back and of the shoulders are very weak, and seem small. The tendon-reflexes in the arms are a little exaggerated; the knee-jerks, the superficial reflexes, and the electrical reactions are normal. The legs are quite unaffected: there is no affection of sensation, of the special senses, or of the bladder or rectum.

The patient's attitude, expression, and the weakness of the back, neck, and shoulder muscles, and some of the other features of his case, suggest the presence of paralysis agitans; but the characteristic tremor and the rigidity of muscles are absent. I am not disposed to attribute the choreiform movements to the patient's former occupation, though the coincidence of the two is noteworthy; and, from his history, he seems to have suffered at one time from a nailmaker's paralysis. The movements seem to have increased, though very slightly, since the onset; and the weakness, though very slowly, steadily progresses. There seemed to be no suspicions of hysteria about the case; the man had never worked in lead; and, on the whole, the case seemed to correspond most with adult chorea.

## Figures and Tables

**Figure f1:**
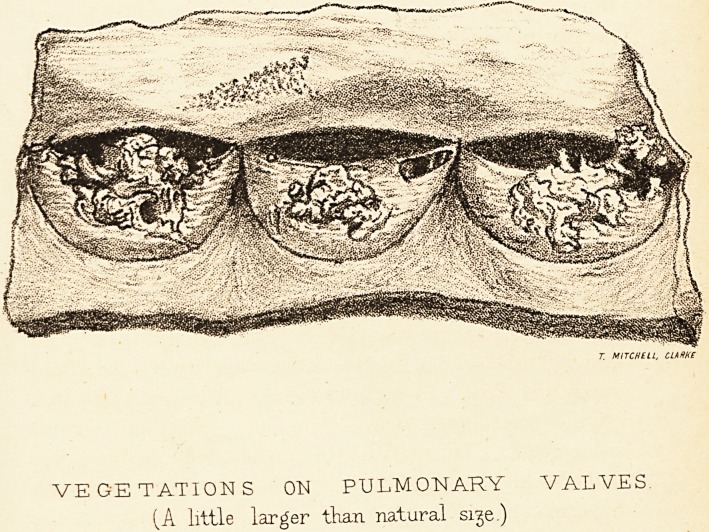


**Figure f2:**